# Lingual Necrosis in the Setting of Giant Cell Arteritis

**DOI:** 10.7759/cureus.74316

**Published:** 2024-11-23

**Authors:** Nivedha Balaji, Aleksandra Ignatowicz, Sandus Khan, Shreya Kuturu, Vaishali Jadhav

**Affiliations:** 1 Department of Internal Medicine, Northeast Georgia Medical Center Gainesville, Gainesville, USA; 2 Department of Internal Medicine, Philadelphia College of Osteopathic Medicine, Philadelphia, USA

**Keywords:** giant cell arteritis (gca), large vessel vasculitis and gca, lingual necrosis, tongue necrosis, vasculitis

## Abstract

Giant cell arteritis (GCA) is a medium-to-large vessel vasculitis most commonly affecting the aortic arch and carotid branches. Lingual necrosis is a rare complication of GCA caused by lingual artery vasculitis due to ischemia. A delay in diagnosis can result in irreversible complications such as tongue amputation. Our case involves an 83-year-old female patient whose hospital admission for evaluation of stroke-like symptoms was complicated by the development of tongue necrosis, requiring percutaneous endoscopic gastrostomy tube placement for severe odynophagia and dysphagia. The patient was treated with IV glucocorticoids; however, she required tongue amputation due to extensive necrosis as a result of delayed initiation of therapy. Treatment of GCA usually involves an initial high-dose steroid with prolonged steroid taper or immunomodulatory, as relapse is a common occurrence. Prompt diagnosis and appropriate therapy to treat GCA is crucial to prevent the progression of tongue necrosis.

## Introduction

Giant cell arteritis (GCA) is an immune-mediated large vessel vasculitis that can manifest as systemic, neurologic, or ophthalmologic complications. Patients may present with scalp tenderness, headaches, jaw or tongue claudication, myalgias, transient ischemic attacks, and vision changes. GCA affects approximately 10 per 100,000 people, with a higher prevalence among Caucasian women over the age of 50 and a peak at 70 years [1,2]. Vascular aging plays a key role in the development of GCA. However, several risk factors, such as atherosclerotic vascular disease, smoking, early menopause, low body mass index, and expression of major histocompatibility complex molecules, particularly human leukocyte antigen, major histocompatibility complex, class II, DR beta 1, have been linked with this disorder [2]. Herein, we present the case of an 83-year-old female patient with a prior history of vascular disease who was diagnosed with lingual necrosis secondary to GCA.

## Case presentation

Our case involves an 83-year-old female patient with a medical history significant for Alzheimer's dementia, carotid artery disease, coronary artery disease, hypertension, and hyperlipidemia who presented to the emergency department with concerns of severe headache, nausea, vomiting, altered mental status, dysarthria, dysphagia, diplopia, and transient left arm weakness of four- to five-hour duration. Vitals on arrival were unremarkable. Laboratory values were significant for white blood cells 14.9 k/uL (normal, 4-10 k/uL), sodium 133 mEq/L (normal, 135-145 mEq/L), and high-sensitivity troponin 213 ng/L (normal, <14 ng/L). The electrocardiogram was negative for ischemia. The physical examination was remarkable for chronic anisocoria, agitation, moderate dysarthria and dysphagia, and normal strength of the upper and lower extremities.

**Figure 1:**
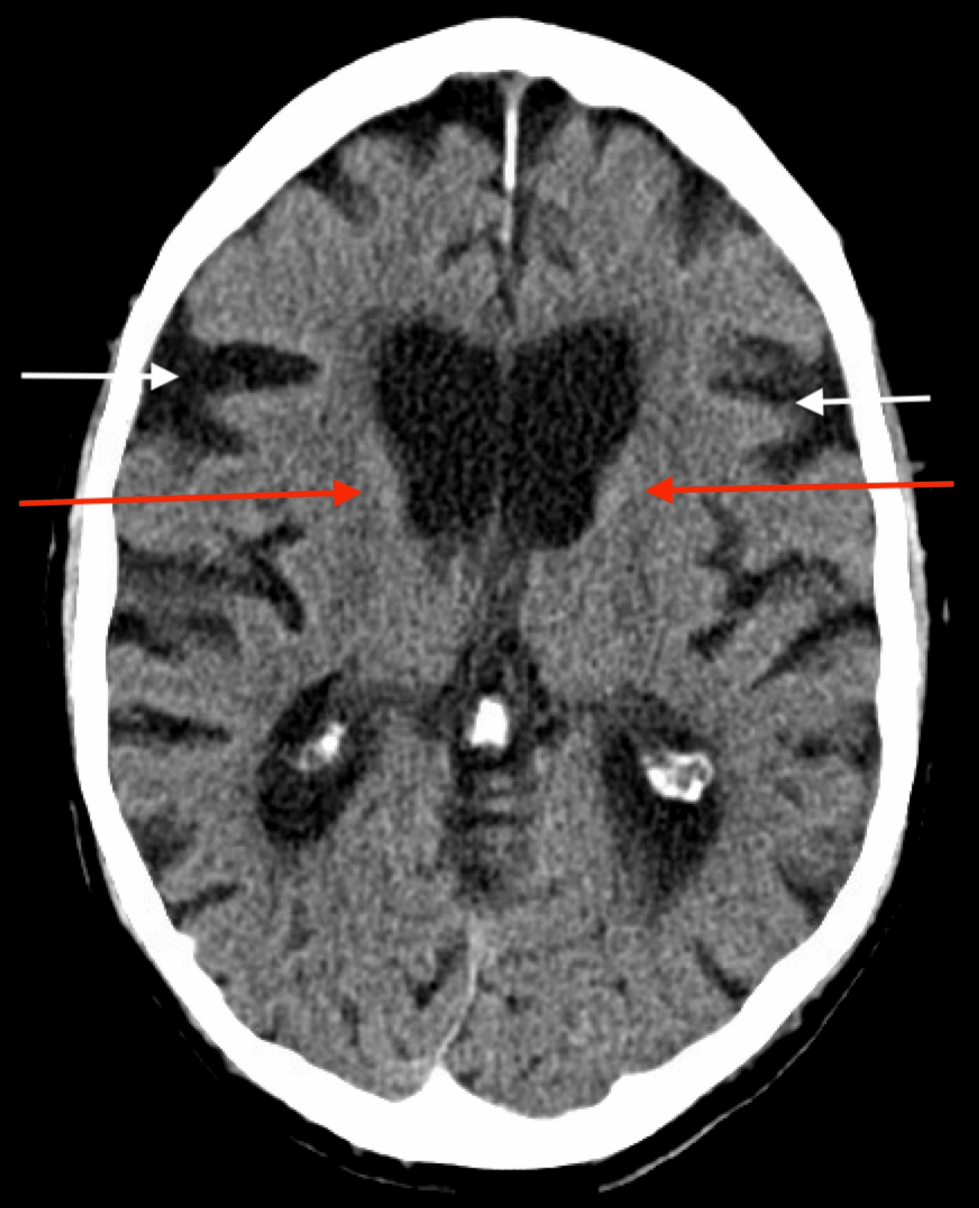
CT of brain, showing cerebral atrophy (white arrows) along with mild periventricular and subcortical microvascular ischemic white matter changes (red arrows) CT: computed tomography

The patient was initially admitted for stroke-like symptoms. Computed tomography (CT) of the brain (Figure 1) noted cerebral atrophy with mild periventricular and subcortical microvascular ischemic white matter changes. Otherwise, CT perfusion and computed tomography angiography (CTA) of the head and neck showed no acute intracranial processes. The echocardiogram did not note any evidence of embolic disease. Further stroke workup was negative.

On day 2 of her hospitalization, the patient developed white-gray discolorations of her tongue, with small lesions and plaques on the right lateral tongue resembling thrush or leukoplakia. Over the next seven days, the lesions progressed to nodular ulcerations with restricted tongue movement and a hyponasal quality to her voice. No ulcerations were noted in the buccal cavity itself. CT maxillofacial showed no obvious soft tissue inflammation or airway narrowing. The patient was treated with oral nystatin solution and fluconazole for glossitis with oral thrush.

**Figure 2:**
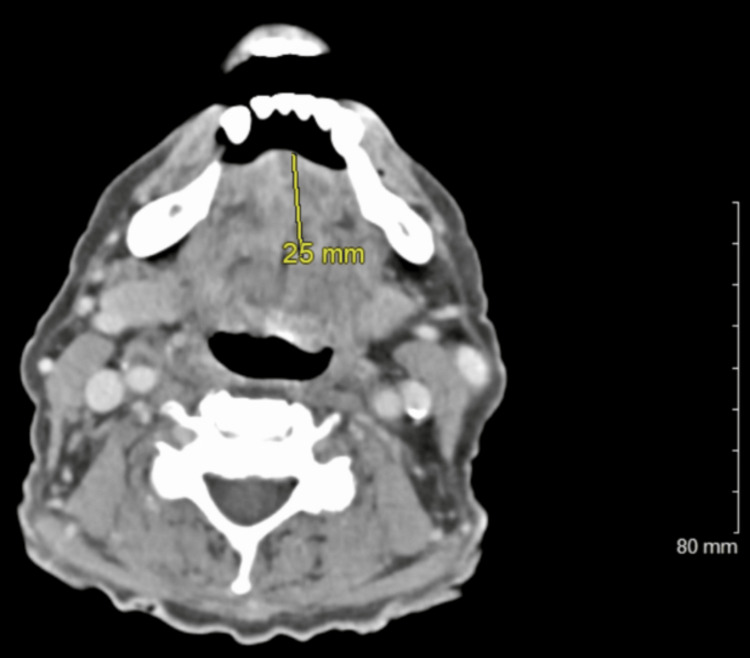
CT soft tissue neck, revealing a 2.5 cm wide heterogenous, necrotic, or cystic enhancement of the anterior tongue CT: computed tomography

**Figure 3:**
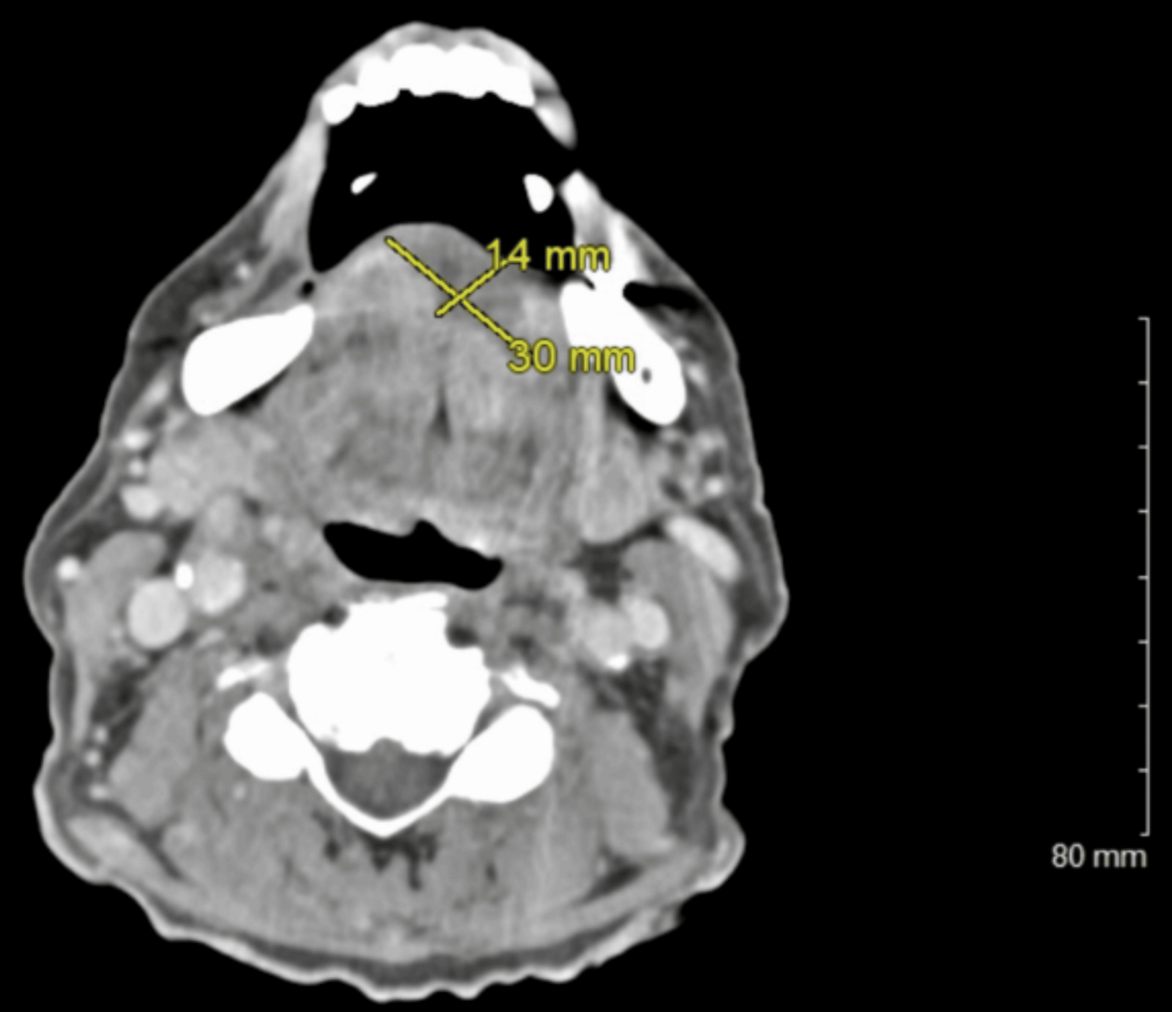
CT soft tissue neck, revealing that the lesion was 1.4 cm in height and 3.0 cm in length and that it extended down to about the floor of the mouth CT: computed tomography

The tongue discoloration transitioned to a diffuse blackish appearance with a large right-sided tongue ulceration after 10 days. Laboratory values noted sediment rate (erythrocyte sedimentation rate, ESR) at 105 mm and C-reactive protein (CRP) at 13.8 mg/dL. A repeat CT soft tissue neck (Figures 2, 3) revealed a 2.5 × 3 cm heterogenous, necrotic, or cystic enhancement of the anterior tongue extending down to about the floor of the mouth concerning a necrotic tumor versus vascular-induced necrosis. There was no focal artery occlusion; however, slightly asymmetrical vascularity was noted with the lingual arteries concerning vasculitis on the CTA head and neck.

Infectious diseases and ears, noses, and throat specialists were consulted due to concerns of tongue necrosis. IV acyclovir was initiated after an oral swab was performed with positive herpes simplex virus 1 (HSV-1) results, and a five-day course of cefepime was completed. Antibiotics were given as there was suspicion of bacterial infection. The patient's family had declined a tongue biopsy at that time.

On day 13 of the hospitalization, a rheumatology service was consulted to rule out GCA and other vasculitis as the underlying etiology of tongue necrosis. The patient was started on high-dose steroids of IV Solu-Medrol 60 mg per day for seven days for clinical suspicion of vasculitis and empiric IV piperacillin-tazobactam for secondary oral infections. CTA of the thoracic aorta revealed mild atherosclerosis without findings of aortitis. The infectious disease specialist recommended the continuation of acyclovir for six weeks as the HSV is known to cause vasculopathy and likely contributed to the progression of tongue necrosis. A temporal biopsy was also recommended; however, the patient's family declined. She tested negative for anti-proteinase 3 and myeloperoxidase antibodies, making granulomatosis with polyangiitis a less likely diagnosis. 

The hospitalization was further complicated by worsening encephalopathy with significant lethargy on day 21. IV steroids were transitioned to oral steroids for possible steroid-induced psychosis, and additionally, acyclovir was also replaced by valacyclovir to reduce the risk of neurotoxicity. The patient's mentation gradually improved, and the tongue necrosis stabilized without any further progression.

**Figure 4:**
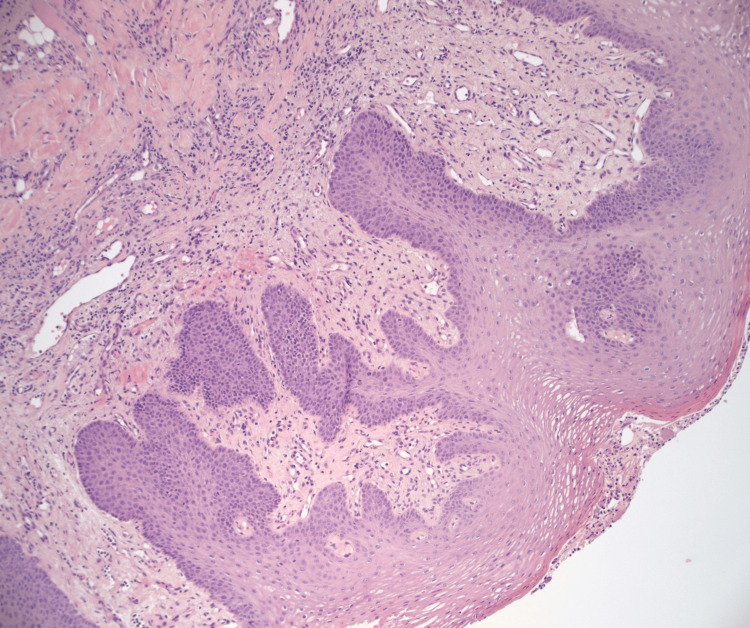
Tissue biopsy revealing ulcerated squamous mucosa without signs of malignancy

**Figure 5:**
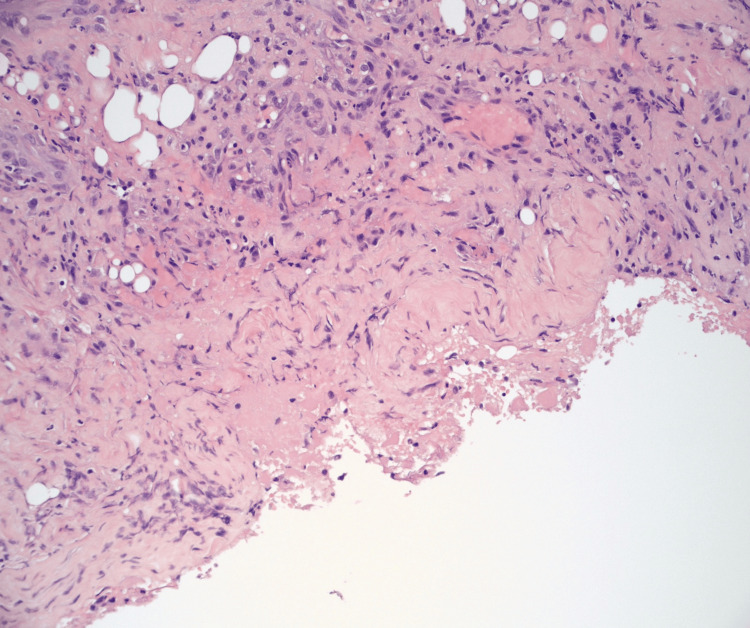
Tissue biopsy revealing ulcerated squamous mucosa with marked reactive changes and chronic inflammation not concerning for malignancy

After approximately four weeks, the family consented to debridement and a tongue biopsy. The biopsy revealed ulcerated squamous mucosa with marked reactive changes and chronic inflammation without signs of malignancy (Figures 4, 5). The biopsy results were consistent with autoimmune conditions such as vasculitis.

The rheumatology specialist prescribed a steroid taper upon discharge, starting at prednisone 60 mg daily. The prednisone dose was decreased by 10 mg every seven days. The option of trialing tocilizumab was discussed with the family; however, they requested to hold off on immunosuppressive therapy. Upon discharge, laboratory values noted improvement in inflammatory markers with ESR at 39 mm and CRP at 1.9 mg/dL. The patient was scheduled to follow up with a Head/Neck Cancer and Reconstructive Surgeon after discharge for a microvascular free flap to maintain her tongue mobility and prevent tethering. The patient was discharged with a rheumatology follow-up for continued management of GCA and steroid taper.

## Discussion

GCA is a form of medium and large vessel vasculitis with a predisposition for the aortic arch and branches of the carotid arteries. However, involvement can also be seen in the iliac and femoral arteries. The disorder is a result of an inflammatory process with transmural infiltration by giant cell granulomas, resulting in luminal occlusion and end-organ ischemia [3]. The disorder manifests with a spectrum of symptoms including fever, temporal headaches, scalp tenderness, jaw claudication, blurred vision, polymyalgia rheumatica, generalized weakness, and amaurosis fugax. Less common complications can include both scalp and tongue necrosis, ischemic stroke, and audiovestibular disturbances. Lingual involvement is not an initial presentation since the tongue is a highly vascularized tissue; however, if the lingual artery is affected, patients endorse tongue edema, pallor, or pain, and later develop tongue ulcerations or necrosis [4]. Tongue necrosis appears to be a delayed finding, affecting approximately 25% of patients, with a majority being elderly women [4,5].

According to the American College of Rheumatology, GCA is diagnosed after fulfilling four of the five criteria: age over 50 years, reduced temporal pulse, new-onset headache, ESR greater than 50 mm/hour, and an abnormal temporal artery biopsy [6]. The histopathology from the biopsy is the gold standard for diagnosis of GCA and is likely to reveal transmural inflammation, multinucleate giant cells, and, in certain cases, focal fibrinoid necrosis. If histology is unavailable, imaging is recommended to support the diagnosis [7]. Other differential diagnoses for the cause of tongue necrosis will need to be ruled out, such as underlying malignancy, medication side effects, radiation and chemotherapy exposure, embolism or hemorrhage, and infectious etiologies like syphilis, herpes, and tuberculosis [5]. Systemic vasculitis such as antineutrophil cytoplasmic antibody-positive vasculitis, most commonly granulomatosis with polyangiitis, can also present with tongue necrosis.

Tongue necrosis and vision loss associated with GCA are irreversible complications that can lead to permanent vision loss or tongue amputation. Therefore, high-dose glucocorticoids should be initiated as soon as GCA is suspected, even before biopsy, if there is strong clinical suspicion. According to the European League Against Rheumatism guidelines, patients with active GCA should be given an initial 40-60 mg/day of prednisone-equivalent dosage immediately to induce remission of active disease [8,9]. In those that exhibit cranial ischemic symptoms, such as amaurosis fugax or vision loss, pulse steroids with IV methylprednisolone 0.25-1 g/day for three days are advised to decrease the long-term prognosis of permanent vision loss. High-dose glucocorticoids should be continued until symptoms have reduced or inflammatory markers have normalized [8,10]. Treatment should be followed with oral steroids at 40-60 mg of oral prednisone daily, which can be adjusted in regard to preexisting health conditions and concerns for steroid side effects [11]. Studies showed that administration of doses higher than 60 mg/day would exceed receptor saturation after several days, resulting in genomic and nongenomic side effects [12,13]. Physicians can then begin a tapering process to reduce the dosages of glucocorticoids to 15-20 mg a day for the next two to three months, followed by 5 mg per day or less after a year [8]. Low-dose steroids alone or with other immunosuppressive agents may be required for one to two years to maintain disease remission and prevent relapse [10]. Relapse is common with GCA as steroids are being tapered; therefore, a prolonged steroid taper is necessary in certain cases. Unfortunately, patients are at risk of glucocorticoid toxicity or have glucocorticoid resistance and are unable to reduce glucocorticoid dose to under 5 mg/day prednisolone equivalent [14]. Subsequently, immunosuppressants are becoming more prevalent for use in the management of GCA.

There is an increasing use of immunosuppressants as adjuvant therapy to minimize total exposure to glucocorticoids as long-term steroid use can lead to higher risks of infection, cardiovascular disease, osteoporosis, osteonecrosis, and diabetes, among other known less-desired side effects [3]. Though research does not support the effectiveness of many immunosuppressants, interleukin-6-inhibitor, tocilizumab, has shown promise in reducing steroid toxicity and relapse rates. According to the Giant-Cell Arteritis Actemra trial, patients treated with tocilizumab at 162 mg weekly or biweekly in addition to a 26-week prednisone taper sustained GCA remission with greater disease control and fewer flares. The patients were also found to have an overall reduction in cumulative prednisone dosage [15]. In the follow-up study that explored the efficacy of tocilizumab 1 year after steroid discontinuation, patients treated with tocilizumab maintained a period of drug-free remission for approximately two years. It was also determined that tocilizumab could be used to treat relapses in addition to prednisone [16,17]. In a study by Cho et al., the patient received a single dose of IV tocilizumab at 6 mg/kg with clinical improvement within several days despite previous steroid therapy resistance. The patient continued monthly tocilizumab infusions while on prednisone tapering dose [4]. However, tocilizumab has not yet been proven to act as a first-line treatment, so its use is recommended to be personalized to patients who have adverse reactions to steroids or who have struggled to taper down their dosage to a safer level. Methotrexate has been studied in multiple clinical trials due to its efficacy in other conditions, such as polymyalgia rheumatica. There is minimal compelling evidence of its addition to conventional steroid use, although several patients were able to taper steroids sooner and reduce overall cumulative steroid dose. The reasoning was attributed to low methotrexate doses used in the studies [17]. There was limited evidence or no benefit found with the use of tumor necrosis factor-alpha inhibitors, such as infliximab and etanercept, or purine analog, azathioprine [14,18,19]. Although tongue necrosis is a rare complication, treatment remains as per GCA guidelines. Most patients respond to glucocorticoid therapy initiation or tocilizumab if there is steroid-resistant lingual necrosis [4,7].

As demonstrated, GCA should be considered as a differential diagnosis with atypical presentation of stroke-like symptoms and tongue ulcerations not improving with antibiotics and anti-viral medications. It is crucial to start appropriate therapy in a timely manner for GCA affecting the lingual artery to halt the ischemia and progression of tongue necrosis. Depending on the extent of the necrosis, patients may need to undergo amputation or surgical debridement of the necrotic area, as our patient did toward the end of her hospitalization [20]. Several cases documented autoamputation of the necrotic region and appropriate reepithelialization after several weeks [5,20]. Our patient also experienced severe odynophagia and dysphagia, ultimately requiring peg tube placement to ensure adequate nutritional status. It is unclear whether the initiation of tocilizumab would have improved the overall prognosis of her tongue necrosis; however, more rapid treatment with glucocorticoids may have delayed the overall damage.

## Conclusions

Diagnosis of GCA may not be immediately apparent resulting in a late recognition and delay in glucocorticoid therapy initiation following the initial presentation. Patients can present with symptoms of scalp tenderness, headaches, jaw or tongue claudication, myalgias, transient ischemic attacks, or vision changes. Treatment with glucocorticoids with or without immunomodulators is necessary to gain control of the disease process, but also to avoid complications or irreversible damage of permanent blindness, tongue amputation, or other ischemic events.
